# Unraveling the interplay: exploring signaling pathways in pancreatic cancer in the context of pancreatic embryogenesis

**DOI:** 10.3389/fcell.2024.1461278

**Published:** 2024-08-22

**Authors:** Sashikanta Swain, Ravi Kant Narayan, Pravash Ranjan Mishra

**Affiliations:** Department of Anatomy, All India Institute of Medical Sciences, Bhubaneswar, India

**Keywords:** pancreatic cancer, transcription factor, signaling pathway, therapeutic approach, embryogenesis

## Abstract

Pancreatic cancer continues to be a deadly disease because of its delayed diagnosis and aggressive tumor biology. Oncogenes and risk factors are being reported to influence the signaling pathways involved in pancreatic embryogenesis leading to pancreatic cancer genesis. Although studies using rodent models have yielded insightful information, the scarcity of human pancreatic tissue has made it difficult to comprehend how the human pancreas develops. Transcription factors like IPF1/*PDX1*, HLXB9, *PBX1, MEIS*, Islet-1, and signaling pathways, including Hedgehog, TGF-β, and Notch, are directing pancreatic organogenesis. Any derangements in the above pathways may lead to pancreatic cancer. *TP53*: and *CDKN2A* are tumor suppressor genes, and the mutations in *TP53* and somatic loss of *CDKN2A* are the drivers of pancreatic cancer. This review clarifies the complex signaling mechanism involved in pancreatic cancer, the same signaling pathways in pancreas development, the current therapeutic approach targeting signaling molecules, and the mechanism of action of risk factors in promoting pancreatic cancer.

## Introduction

The close association between the incidence and death highlights the terrible prognosis of pancreatic cancer (PC). Recent data indicates that the 5-year survival rate for pancreatic cancer worldwide is approximately 11% (Pancreatic Cancer Statistics, 2024). Adding to this low survival rate is late late-stage diagnosis of the disease (Kamisawa et al., 2016). Currently, PC is the seventh leading cause of cancer-related deaths worldwide (Rawla et al., 2019) and is assumed to become the second major cause of death globally by 2030. The European countries have the highest occurrence rates, and China leads the Asian nations in incidence and fatality, followed by Bhutan, Nepal, and India (Gaidhani and Balasubramaniam, 2021; Kamisawa et al., 2016; Raimondi et al., 2009).

PC is more common in men (5.5 per 100,000, 243,033 cases) than in women (4.0 per 100,000, 215,885 cases). PC is more commonly observed in elderly populations and rarely diagnosed before 55 years of age. The treatment strategies are complicated due to early metastasis, recurrence, and resistance to radiation and chemotherapy (Brunner et al., 2019). Signaling pathways play a significant role in disease progression and behavior. When signaling pathways malfunction, cancer develops. Cancerous cells can grow and metastasize due to mutations and dysregulation in the signaling pathways that link with pancreatic development (Kamisawa et al., 2016).

Understanding the signaling pathways in pancreatic development is crucial for comprehending PC. In recent years, research on transcription factors involved in the development, function, and disease process of the pancreas has expanded (Jennings et al., 2020). These include the signaling mechanisms that might regulate cell connections in the developing pancreas. Here, we will explore the factors and genes involved in the signaling pathways leading to PC while exploring how these pathways are involved in pancreatic embryogenesis.

### Signaling pathways in pancreatic morphogenesis and cancer

**FIGURE 1:**
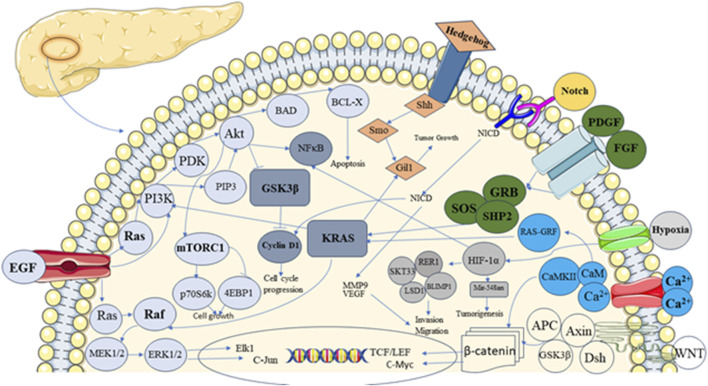
Showing different signalling pathways involved in pancreatic development and carcinogenesis.

The pancreas is a unique tissue with endocrine and exocrine components. Acinar and duct cells constitute the exocrine portion of the pancreas, and the islet of Langerhans comprises endocrine components (Rizk et al., 2023). Ghrelin, somatostatin, insulin, glucagon-producing cells, and pancreatic polypeptide comprise the endocrine part of the islet of Langerhans (Ornellas et al., 2020; Murtaugh and Melton, 2003). The development of endocrine and exocrine cells, along with acini formation, is influenced by signaling pathways (Dohrmann et al., 2000). Signaling pathways are complex networks of molecular interactions that allow cells to communicate with each other and respond to external signals (Azeloglu and Iyengar, 2015). Interaction between exocrine and endocrine components of Langerhans through signaling components results in controlled hormone secretion. The signaling component involves ligands, receptors, and intracellular Smads (Figure 1), which are present in the pancreatic epithelium and mesenchyme (El-Gohary et al., 2013).

Pancreatic embryogenesis requires complex signaling pathways; Insulin Promoter Factor 1/Pancreatic and Duodenal homeobox 1 (IPF1/*PDX1*) is the most significant transcription factor, the earliest expressed in the embryonic pancreas. IPF1/*PDX1* is a ParaHox group homeodomain transcription factor essential for the development of the pancreas in humans and mice (Rosanas-Urgell et al., 2005). This signaling molecule is expressed in pancreatic cells of postnatal mice, and literature indicates that this signaling pathway is necessary for specific tasks in the mature cell (Ebrahim et al., 2022). It responds to growth signals from the mesenchyme (Kim and MacDonald, 2002) and is expressed in the endoderm. The expression is not limited to pancreatic tissue, as *PDX1* mutant mice commence dorsal and ventral bud development (Offield et al., 1996), indicating that other components are needed to designate the pancreas anlage. The IPF1/PDX1 pathway is dysregulated in PC, leading to a loss of normal functions. The loss of IPF1/*PDX1* function can lead to abnormal cell growth and decreased differentiation of pancreatic cells, creating an environment conducive to cancer development (Roy et al., 2016). IPF1/PDX1 regulates downstream molecules such as Neurogenin 3 (NGN3), Forkhead Box A2 (FOXA2), Hepatocyte Nuclear Factor 1 Beta (HNF1B), Fibroblast Growth Factor Receptor 2 (FGFR2IIIB), and Spondin 1, which are involved in pancreatic development, differentiation, and function in pancreatic cancer (Svensson et al., 2007; Oliver-Krasinski et al., 2009). IPF1/*PDX1* expression levels are also being explored as potential diagnostic and prognostic markers for PC (Kim and MacDonald, 2002). The tumor’s aggressiveness can be determined based on IPF1/PDX1 expression changes. Changes in signaling pathways, epigenetic modifications, and genetic mutations might cause dysregulation in IPF1/*PDX1* expression (Kim and MacDonald, 2002).

Pre-B-cell leukemia homeobox- 1 (*PBX1*) and Myeloid Ecotropic Integration Site (*MEIS*) regulate the DNA binding activity of other gene products like IPF1/*PDX1*. They control gene expression and coordinate pancreas development (Kim and Hebrok, 2001). *PBX1* and *MEIS* interact with *HOX* genes and form complexes that regulate gene expression during the embryonic development of the pancreas. Dysregulation of these interactions can potentially affect the normal growth and maintenance of pancreatic tissue, indirectly contributing to the initiation or progression of PC (Girgin et al., 2020). *MEIS1* mutant mice die during embryogenesis from hematological and vascular abnormalities, indicating that *MEIS1* is crucial for embryonic development (Burstin et al., 2010). *PBX1* contributes to cancer by affecting lineages within the hematopoietic system, such as B cells, HSCs, and Mk-Erythrocyte Progenitors (MEP) (Muggeo et al., 2021).

Similarly, Homeobox gene B9 (HLXB9) is a vital transcription factor required for dorsal pancreatic development and the formation of insulin-producing beta cells (Jensen J, 2004). HLXB9 altered expression contributes to the progression of the disease. Still, it is not a driver mutation in PC; HLXB9 expression can impact the cellular environment and promote cancerous growth (Chen et al., 2018). In order to maintain cancer cell proliferation, HLXB9 upregulates genes that are involved in the G1-S transition of the cell cycle, such as Cyclin E1 (CCNE1) and Cyclin E2 (CCNE2) (Chen et al., 2018). Understanding HLXB9’s role in pancreatic development could have implications for regenerative medicine and potential therapies that aim to restore or repair pancreatic tissue in patients with PC (Desai et al.,2015). Hence, a temporally prolonged expression of HLXB9 leads to severe impairment of pancreatic development, whereas a total loss of HLXB9 expression blocks the start of the dorsal pancreatic program (Li and Edlund, 2001).

Transforming growth factor - beta (TGF-β) signaling components, including ligands like activin and TGF-β and their corresponding receptors, as well as ligand antagonists like follistatin, noggin, and gremlin, are present in both mesenchyme and epithelium of embryonic and adult pancreas (Chang, 2016). The distinct functions TGF proteins play in regulating the endocrine and exocrine pancreas are suggested by the varied expression patterns for each TGF-β isoform. All of them are expressed persistently and become localized to the acinar cells later in gestation. They are all faintly present in the E12.5 epithelial cells early in the pancreas formation (Rane et al., 2006).

Additionally, TGF-β signaling regulates several cellular functions; dysregulation of this signaling results in the onset and spread of cancer (Baba et al., 2022). Upon binding of active TGF-β to a class of transmembrane serine-threonine kinases known as Type I and Type II TGF-β receptors (TβRI and TβRII, respectively), the TGF-β signaling cascade is initiated. (Aashaq et al., 2022). After binding to TβRII, the TGF-β ligand recruits TβRI to form a complex. TβRII can cross-phosphorylate TβRI, which activates it, thanks to this ligand-bound receptor complex (Smith et al., 2012). In advanced stages, TGF-β can promote tumor growth and metastasis. However, In the early stages of cancer, it acts as a tumor suppressor by inhibiting cell growth and promoting apoptosis (Liu et al., 2021). A similar trend is for PC; TGF-β signaling often becomes dysregulated, contributing to disease progression. TGF-β can function as a tumor suppressor by inhibiting the uncontrolled proliferation of cancer cells in early pancreatic carcinogenesis (Sabbadini et al., 2021). Early stages of PC development are due to the loss of the TGF-β receptor or mutations in downstream signaling components that disrupt this tumor suppressor role (Truty and Urrutia, 2007). The TGF-β signaling pathway is the current research interest because of its dual nature. TGF-β signaling inhibitors are being investigated as possible therapeutics to counteract TGF-β′s pro-tumorigenic activities and re-establish its tumor-suppressive properties (Javle et al., 2014).

Early on, it was discovered that follistatin was widely distributed in pancreatic mesenchyme and vanished at the E12.5 stage of pancreas development. Follistatin is still expressed in adult islets and reappears in E18.5 (Maldonado et al., 2000). Throughout the early stages of embryogenesis, mesenchyme-derived follistatin inhibits epithelium-derived activin, allowing for unopposed exocrine differentiation and a relative reduction in endocrine differentiation (Namwanje and Brown, 2016). Epithelial activin is released later in life due to a decrease in mesenchyme compared to epithelium and a drop in follistatin levels (Xia and Schneyer, 2009). This helps to differentiate endocrine cells into mature islets after birth (Maldonado et al., 2000). TGF-β antagonist follistatin is a glycoprotein primarily known for regulating the activity of TGF-β family members, such as activin and myostatin. The ability of follicular fluid to block follicle-stimulating hormone (FSH) led to the discovery of follistatin (FST) (Kappes et al., 2023; Welt et al., 2002). Activins are bound to FST, a monomeric glycosylated protein, with remarkable affinity, neutralizing their binding affinity and bioactivity. FST also exhibits a lower affinity for binding to myostatin (MST) and Bone Morphogenetic Proteins (BMPs) 2, 5, 7, and 8 (Sidis et al., 2006; Amthor et al., 2004; Hedger et al., 2011; Iemura et al., 1998) in addition to other TGF-β superfamily members. These studies demonstrate how FST may affect the biological activities of many members of the TGF-β superfamily, especially at greater doses (Pervin et al., 2021). Follistatin inhibits TGF-β signaling pathways by binding to ligands and preventing them from binding to their receptors (Harrington et al., 2006). It affects embryonic exocrine and endocrine cell differentiation. By controlling the TGF-β pathway, follistatin shapes cell development in the pancreas (Lee et al., 2021; Kahata et al., 2018).

Similarly, pancreas transcription factor 1A (PTF1A) plays a crucial role in controlling the proliferation of multipotent progenitor cells throughout pancreatic development and in the maintenance and specification of acinar cells. The inhibitory neuronal cell fate in neural tissues is determined by PTF1A, transiently produced in the post-mitotic cells, and mediated mostly by downstream genes such as transcription factor activation profiles (TFAP2A/B) and Positive Regulatory Domain (PRDM13). In humans and rodents, Mutations in the coding and non-coding regulatory spaces cause PTF1A gain or loss of function, which is linked to hereditary disorders such as pancreatic and cerebellar agenesis (Jin and Xiang, 2019).

The vital pancreatic transcription factor PTF1A is induced in the dorsal pancreatic endoderm by aortic endothelial cells; in contrast, ventral PTF1A induction and ventral pancreatic bud commencement, do not require the vitelline veins, which are often located next to the developing ventral pancreatic bud. We discover that PTF1A is induced in dorsal endoderm explants by the aorta cells, independent of the blood supply (Yoshitomi and Zaret, 2004).

The Sry-related HMG box (SOX) family of transcription factors, also known as the sex-determining region on the Y box, is involved in developing multiple tissues throughout embryogenesis and determining cell destiny. In *PDX1+* ductal cells of the human pancreas, SOX9 is detected in the eighth week of embryonic development. It frequently co-localizes with Neurogenin-3 (NGN3) and other significant islet beta-cell progenitor markers (McDonald et al., 2012). SOX9 transcription factor plays a less important role in the development of the exocrine pancreas and regulates the adoption of an endocrine phenotype (Seymour et al., 2008).

### Notch signaling in pancreatic embryogenesis and cancer

Tumor-suppressing and tumor-promoting effects in PC are mediated by Notch signaling. Cancer cell proliferation, metastasis, and cancer stem cell phenotype formation are also mediated by notch signaling. Notch signaling governs the development of pancreatic endocrine and exocrine cells. Notch signaling involves the activation of ligands by neurogenin genes, which leads to the transcription of Hairy and Enhancer-of-Split (*HES)* genes that influence cell fate (Kim et al., 2010; Li et al., 2016). Abnormalities in Notch signaling and PC are associated. The most common PC, pancreatic ductal adenocarcinoma (PDAC), has the notch pathway dysregulated. Several studies have shown that NGN3, a pro-endocrine factor, is negatively regulated by Notch signaling. Pro-endocrine factor activation or Notch processing inhibition dramatically promotes the development of insulin-producing β-cells. However, as of late, several scientists have disputed that the Notch pathway prevents the growth of endocrine cells. It has been suggested that either the inactivated Notch pathway favors acinar cell development or the Notch pathway determines the pancreatic progenitors developing towards endocrine lineage. Based on the current research, Notch signaling controls the quiescence, self-renewal, and differentiation of pancreatic progenitor cells during pancreatic development in a manner dependent on the Notch level (Li et al., 2016).

### Hedgehog (Hh) signaling in pancreatic embryogenesis and cancer

Hedgehog signaling, in addition to notch signaling, is essential for PC. Hh ligands (Sonic, Indian, and Desert Hhs), Smoothened (SMO), Patched receptor (PTCH1), and transcription factors (GLI1, GLI2, and GLI3) are essential elements of the Hh signaling system. The Hh ligands bind to the transmembrane receptor, PTCH1, which usually inhibits another transmembrane protein, SMO. Active SMO triggers a downstream signaling cascade that involves a family of transcription factors known as GLI proteins (GLI1, GLI2, and GLI3). In the absence of Hh signaling, GLI proteins are inhibited through a complex formation involving other proteins, resulting in abnormal cell growth. Activin signaling suppresses the expression of Sonic Hedgehog (SHH), ensuring proper pancreatic development (Han et al., 2016). The differentiation of diverse cell types in the embryonic pancreas is then regulated by Hh signaling pathways (Carballo et al., 2018). The Hh pathway regulates insulin production in the adult pancreas, but it is also necessary to regenerate the exocrine pancreas in response to damage, Its activity is highly restricted to the beta-cells of the endocrine pancreas.

Two independent studies first reported aberrant activation of the Hh pathway in human PC. The normal pancreas does not produce Shh, whereas 70% of human PC samples exhibit overexpression of Shh in both pre-invasive and invasive epithelium; this overexpression can be observed as early as pancreatic intraepithelial neoplasia-1 (PanIN1) and persists throughout the disease (Gu et al., 2016). On the other hand, most PC cell lines have an abnormal expression of the Hh ligand. This finding in PDAC in humans was also validated in a genetically modified mice model. In PDAC, oncogenic Kirsten rat sarcoma (*KRAS)* expression is closely linked to abnormal SHH expression. Increased SHH transcript results from oncogenic *KRASG12D* ectopic expression in healthy human pancreatic ductal cells, suggesting that SHH functions as a downstream effector of oncogenic *KRASG12D* in developing PC. It has also been demonstrated that NF-κB targets the gene SHH and is constitutively activated in PC. In both cell-based and *in-vivo* scenarios, NF-κB activation can enhance SHH’s transcriptional activity. The putative NF-κB binding sites are present in the human SHH promoter region. Furthermore, oncogenic KRAS is recognised to activate the transcriptional activity of NF-κB. Thus, oncogenic KRAS may use NF-κB signaling to encourage SHH expression (Gu et al., 2016).

### Oncogenes altering common signaling pathways

The literature presents an increasing number of oncogenes that cause PC (Hezel et al., 2006; Avila and Kissil, 2013; Wood, 2013). When oncogenes are mutated and activated, they contribute to cancer growth. PC was attributed to genetic alterations, germline mutations, and somatic mutations. *KRAS, TP53, CDKNA2A, MLL3, ZIM2, MAP2K4, ARID1A, NALCN, SMAD4, EPC1, ARID2, ATM, TGFBR2, SLC16A4, SF3B1*, and *MAGEA6* are the sixteen mutant oncogenes that have been found majorly associated with this disease (Cicenas et al., 2017).

*KRAS* mutations, one of the earliest changes in PC, trigger signals promoting cancer cell survival and multiplication (Berrozpe et al.,1994). These mutations lead to the continuous activation of the KRAS protein, which acts as a molecular switch to activate several downstream signaling pathways, including the MAPK/ERK and PI3K/AKT pathways (Buscail et al., 2020). Tumor suppressor genes help control cell growth and prevent tumors from forming. In PC, these brakes often malfunction due to mutations (Bardeesy and DePinho, 2002). *P16/CDKN2A, TP53*, and *SMAD4/DPC4* are critical players in this process. Loss of P16/CDKN2A function leads to unchecked cell cycle progression from the G1 to the S phase. Loss of SMAD4 function disrupts TGF-β signaling and promotes epithelial-mesenchymal transition (EMT), enhancing the invasiveness and metastatic potential of pancreatic cancer cells (Hu et al., 2021). Loss of these genes removes the brakes, giving cancer cells a growth advantage (Austen and Bronte, 2023).

Growth factor receptors work as antennas on the cell surface. These antennas receive signals from growth factors like epidermal growth factor (EGF), insulin-like growth factor (IGF), and vascular endothelial growth factor (VEGF). In PC, growth factor receptors can be proactive, leading to uncontrolled cell growth and spread. When activated, the receptor for advanced glycation end products (RAGE) acts as a magnifier of inflammation and promotes the progression of PC. Researchers are exploring ways to turn down the activity of RAGE to slow down cancer growth and enhance the effectiveness of treatments (Faruqui et al.,2022). EMT is crucial for cancer cells to spread and form new tumors in distant places (Zhou et al., 2017). The WNT signaling pathway acts as a control center for cell growth (Scheibner et al., 2019). This control center can go haywire in PC, leading to unregulated cell division and migration (Zeng et al., 2006).

### Risk factors and their mechanism of action in altering signaling pathways

Risk factors like the combination of family history, obesity, smoking, diabetes, and chronic pancreatitis can contribute to the development of PC. A genetic predisposition to the disease is noticed by studying a family history of PC. Hereditary mutations in the *BRCA1, BRCA2*, and *PALB2* genes are risk factors for PC. These genetic alterations may affect cellular signaling pathways, including *KRAS* oncogene activation. Similarly, Genetic mutation can also cause disruptions to cellular processes, potentially leading to the activation of WNT/β-catenin Pathway and an increased risk of developing PC (Zanini et al., 2021).

One of the most common risk factors that raises the possibility of developing PC is type 2 diabetes (Pannala et al., 2008). The complete mechanism is still a doubt for researchers. However, there is evidence that insulin resistance and abnormal signaling pathways, such as the serine/threonine kinase (AKT) pathway, may play a role. AKT (AKT1, AKT2, AKT3), previously known as protein kinase B (PKB), signaling pathway is currently driving the research as it plays a vital role in primary cellular functions, including regulation of glucose metabolism, cell size, and cell cycle progression (Hu et al., 2022; Permert et al., 1993). In type 2 diabetes Insulin resistance and hyperinsulinemia activate PI3K/AKT pathway, low grade inflammation activates the NF-κB pathway and high glucose levels activate the TGF-β1 pathway, leading to a decrease in E-cadherin levels and promoting a mesenchymal phenotype (Gong et al., 2014; Duan et al., 2021).

Another risk factor for PC is Chronic pancreatitis. Chronic inflammation leads to the activation of Pancreatic Stellate Cells (PSCs), which transform into myofibroblast-like cells. PSCs secrete chemokines, reactive oxygen species, and cytokines which activates TGF-β, MAPK, and NF-κB, promoting cancer cell proliferation, invasion (Jin et al., 2020).

There is an established connection between the risk of PC and obesity. Adipose tissue secretes leptin, and it is possible that leptin could influence Hh signaling (Zanini et al., 2021). Like in other cancers, smoking is a well-established risk factor in PC. nitrosamines and polycyclic aromatic hydrocarbons present in smoke, can cause mutations in the KRAS gene and activates NF-κB Pathway, Overall leads to mutation in key genes like KRAS, p53, and CDKN2A. Nicotine, a major component of tobacco smoke, can bind to nicotinic acetylcholine receptors (nAChRs) on pancreatic cells. This binding activates the EGFR signaling pathway, leading to increased cell proliferation and survival (Schaal and Chellappan, 2014; Weissman et al., 2020). Accumulation of mutations and epigenetic alterations in the DNA due to age-related changes can lead to the activation of oncogenes or the inactivation of tumor suppressor genes. Gender-related differences may influence oncogene activation by triggering hormonal and genetic factors. For instance, hormones, including estrogen, testosterone, and insulin-like growth factor-1 (IGF-1) in men, significantly contribute to a higher incidence of PC. Genetic variations between racial groups lead to oncogene activation differences (Zanini et al., 2021).

### Therapeutic interventions targeting common pathways

The initial step in treating PC is to remove the tumor surgically, which is followed by gemcitabine-based chemotherapy. In cases where patients exhibit a favourable performance status and surgery is not feasible, a combination of gemcitabine, FOLFIRINOX, and nanoparticle-bound (nab) paclitaxel is used. However, the prognosis is still dismal, and chemotherapy medications have only been shown to be palliative in PC patients whose cancer has spread or is incurable (Lambert et al., 2019). However, there is hope due to the known molecular mechanisms underlying the onset and spread of PC and the availability of novel medications that can disrupt essential signaling pathways (Polireddy and Chen, 2016).

Another therapeutic target for PC can be the hypoxia-inducible factor (HIF1α), which is a downstream effector of PBX1-MEIS1. This target utilisation has been observed to influence Myeloproliferative neoplasm (MPN) cells via PBX1 (Crisafulli et al., 2024). As discussed, HLXB9 can play a significant role in tumor progression, the GSK-3β phosphorylates and stabilizes the HLXB9 protein and, therefore, can be targeted to control the development of insulinomas (Desai et al., 2014).

Many inhibitors have applications in treating PC since the PI3K/AKT pathway can be blocked at multiple places (Stanciu et al., 2022). The mTOR kinase inhibitors, such as everolimus, are among the medications used to slow the disease progression to an end-stage and can also improve the effectiveness of gemcitabine-based chemotherapy. Agents that have demonstrated enhanced efficacy and can inhibit mTORC1 and mTORC2 are preferable. By attaching to the PH domain of AKT, perifoxine (KRX041, NSC639966) functions as an allosteric AKT inhibitor (Glaviano et al., 2023). Numerous clinical trials with this alkyl phospholipid have been conducted following encouraging outcomes from studies conducted on animal models. In pancreatic cell cultures, erifosine suppresses S6K1–GLI1 signaling and prevents gemcitabine resistance (Ying et al., 2014). Prior research indicates that cancer stem cells play a significant role in patient relapse, maybe through the reactivation of the SHH signaling pathway and the PI3K/AKT/mTOR pathway. A combination of NVP-BEZ235 and NVP-LDE225 may provide new hope for treating PC (Sharma et al., 2015). An oral medication called NVP-LDE225 (Sonidegib, Novartis) inhibits the Hh pathway by acting as an antagonist for the SMO receptor (Pan et al., 2010; Ruiz-Borrego et al., 2019; Wang et al., 2019; Stanciu et al., 2022).

Anti-TGF-β treatments are effective in preclinical research, and several of these tactics are presently undergoing clinical trials. TGF-β neutralizing antibodies, which prevent TGF-β ligands from binding to their receptor, TGF-β receptor kinase inhibitors, and direct delivery of antisense oligonucleotides (ASO) into tumors or immune cells are the three main strategies for inhibiting TGF-β or its pathway components. (Smith et al., 2012).

Although anticancer drugs have historically focused on TGF-β pathway inhibitors, the efficacy of contemporary therapy has not kept pace with this focus (Tian et al., 2019). This emphasizes the need for a more thorough investigation of FST as a strong and efficient TGF-β antagonist that can target cancer cells specifically or stop them from developing resistance to TGF-β′s anti-proliferative effects. It can also be used as a biomarker to classify cancer patients and enhance their responses to treatment more accurately. Comprehending the role of FST in specific disorders holds the potential for creating innovative therapeutics, especially with the current focus on secreted molecules in drug development (Table 1) (Sosa et al., 2024).

**TABLE 1:** Signaling pathways involved pancreas embryogenesis, pancreatic cancer and therapeutic development targeting the same signaling pathways.

Signaling pathway	Role in pancreas development	Role in pancreatic cancer	Therapeutic approach	Clinical trial drugs
K-Ras	Involved in cell signalling, cell growth, and differentiation. Specifying pancreatic progenitor cells from the endodermal tissue during pancreas development. (Hingorani et al., 2003)	It is frequently mutated in pancreatic ductal adenocarcinoma (PDAC). It drives tumor growth, invasion, and metastasis. (Bryant et al., 2014; Luo, 2021; Strickler et al., 2023)	Small-molecule inhibitors targeting K-Ras membrane localization (e.g., FTIs, GGTIs), SOS/K-Ras interactions, and downstream effectors are under investigation; (Polireddy and Chen, 2016; O'Bryan, 2019)	ELI-0027P (NCT05726864) Sotorasib (AMG 510) (NCT03600883). and Adagrasib (MRTX849) (NCT03785249) both specifically targets the KRAS G12C mutation. MRTX1133 (NCT05737706) targets KRAS G12D mutation (Bannoura et al., 2021)
Histone deacetylase	Critical for epigenetic regulation of gene expression; Histone deacetylase (HDAC) deregulation implicated in pancreatic cancer development. (Klieser et al., 2015; Haumaitre et al., 2008)	Overexpression of specific HDAC isoforms observed in pancreatic cancer; HDAC inhibitors show anti-tumor effects in preclinical models. (Hai et al., 2021; Li et al., 2020)	HDAC inhibitors (HDACi) tested include SAHA, romidepsin, valproic acid, and entinostat; ongoing research focuses on selective HDAC inhibitors. (Safari et al., 2023; Xiang et al., 2022)	pan-HDACi CG200745 (NCT02737228) (Wu et al., 2020)
Hh signaling pathway	Hh signaling is significantly involved in growth regulation and embryonic patterning. (Hebrok et al., 2000)	The ligand-dependent activation of the Hh pathway is more significant in carcinogenesis. (Onishi and Katano, 2014; Kayed et al., 2004)	Natural compounds like epigallocatechin-3-gallate and sulforaphane inhibit pancreatic CSCs via Hh signaling suppression. Smo inhibitors (e.g., GDC-0449, IPI-926, LDE225) and Gli transcription factor inhibitors (e.g., GANT-61) target Hh signaling for anti-tumor effects; (Quatannens et al., 2022; Onishi et al., 2022; Nguyen and Cho, 2022; Huang et al., 2013)	Adjuvant Autogene Cevumeran Plus Atezolizumab and mFolfirinox Versus mFolfirinox (NCT05968326), Taladegib (NCT05199584) in phase-2 clinical trial; (Xie et al., 2019, Sally et al., 2022)
Notch Signaling	Notch was initially expressed in pancreatic epithelial cells at E9.5, and later by E14.5, it was broadly expressed in the pancreatic epithelium. Specific Notch pathway component silencing promotes premature endocrine pancreatic development. (Li et al., 2016)	Notch signaling can activate genes involved in cell cylcle, which leads to increased proliferation. Hes1, the Notch target, is expressed more in PanIN lesions than in normal ducts, according to an analysis of the Pdx1-Cre; LSL-KrasG12D mouse model. (Avila and Kissil, 2013)	Gamma secretase inhibitors (GSIs) like MRK-003 and PF-03084014 were tested in preclinical and clinical studies. (You et al., 2023; Fang et al., 2023)	RO4929097 (NCT01196416) clinical trial completed in 2015. Aderbasib (NCT04295759) is in progress for glioma (Li et al., 2023; You et al., 2023)
Cancer Stem Cells (CSCs) signaling pathway	Embryonic stem (ES) cells have been able to differentiate into pancreatic and endoderm lineages either through prolonged (up to 4 weeks) cell culture manipulations or through overexpression of transcription factors such as HNF3β (Wells JM, 2003)	CSCs can spread tumors, and become resistant to radiation and chemotherapy. Through activating Hedgehog, Wnt, Notch, JAK-STAT, Nodal/Activin, and Hippo pathways. CSCs mediate tumor induction and proliferation. (Bubin et al., 2023; Barman et al., 2021)	Clinical trials for monoclonal antibodies (e.g., NPC-1C) and natural compounds (e.g., curcumin, resveratrol) targeting CSCs in preclinical models are ongoing. (Lo Iacono et al., 2022; Hashem et al., 2022)	NALIRIFOX Plus Radiation Therapy (NCT05851924) Napabucasin (NCT02178956), MCLA-128 (NCT02912949) targets STAT3 signaling. (Ahn et al., 2018)
PI3K pathway	Cellular rearrangements between acinar and ductal cells are regulated by the IGF/PI3K pathway. It regulates the protrusion and rearrangement of epithelial cells associated with morphogenesis. (Darrigrand et al., 2024)	In 5% of pancreatic cancer cases, there has been an incidence of mutation in genes encoding the PI3K pathway, particularly in PI3KCA (encoding the p110α subunit of PI3K); these mutations result in activation of the PI3K pathway, (Buchanan et al., 2015; Noorolyai et al., 2019; Vara et al., 2004)	Various inhibitors targeting the PI3K pathway, including mTOR kinase inhibitors (e.g., everolimus) are under investigation. (Glaviano et al., 2023; Sirico et al., 2023)	ASP2138 (NCT05365581), EO-3021 (NCT05980416), AZD0901 (NCT06219941) (Yang et al., 2019; Stanciu et al., 2022)
Hypoxia	Involved in beta cell damage. (Gerber and Rutter, 2017)	Hypoxia-induced Hh signaling promotes EMT; promoting tumor development. Human PDACs are highly hypoxic. (Li et al., 2021; Yuen and Díaz, 2014)	Hypoxia activates Hh signaling independently of HIF-1α, representing a therapeutic target; combination therapies with Hh inhibitors and gemcitabine or cisplatin show synergistic effects; (Onishi et al., 2013; Gu and minko, 2024; Rocha et al., 2018)	TH-302 (Evofosfamide) (NCT02076230) targets hypoxic tumor cells and PX-478 (NCT00522652) acts by inhibiting hypoxia-inducible factor-1 alpha (HIF-1α). (Tao et al., 2021; Kao et al., 2023)
NF-κB Signaling Pathways	The balance between β-cell proliferation and apoptosis throughout the early stages of beta cell development is maintained by NF-κB through the physiological regulatory circuit. In the T1D mouse model, NF-κB controls the β-cells and the progression of diabetes. (Sever et al., 2021)	In pancreatic cancer, NF-κB is actively expressed due to oncogenic Kras mutations and inflammatory signaling pathways. NF-κB activation in PDAC is influenced by chemokines, cytokines, and chronic inflammation and it controls molecules related to angiogenesis and metastasis. (Prabhu et al., 2014; Silke and O’Reilly, 2021)	Phytochemicals like curcumin and COX inhibitors can inhibit NF-κB and other signaling pathways, offering potential therapeutic benefits in PDAC. (Pramanik et al., 2018; Ebrahimi et al., 2024)	AVA6000 (NCT04969835), Bortezomib (NCT01668719) inhibits the proteasome, which inhibits NF-κB and has shown potential in early-phase clinical trials (Chen et al., 2011)
EGFR	Pancreatic acinar and ductal cells differentiate into endocrine islet cells via EGFR. Therefore, EGFR is crucial for controlling β-cell bulk. (Miettinen et al., 2008)	Overexpression of EGFR or the ability of mutant versions to control downstream signaling is observed in pancreatic cancer. (Fitzgerald et al., 2015; Oliveira-Cunha et al., 2011)	By competing with one another for receptor binding, anti-EGFR antibodies (Panitimumab, Cetuximab) prevent ligand-induced autophosphorylation. Small molecule inhibitors (Erlotinib, Gefitinib) compete with ATP for binding to the EGFR tyrosine kinase’s intracellular catalytic domain. (Fang et al., 2023; Kung and Yu, 2023; Giovannetti et al., 2008)	DS-1062a (NCT03401385), RC68 (NCT05383547) based Antibody-Drug Conjugates (ADCs) shown promise in preclinical studies and early-phase clinical trials. (Li et al., 2019).
VEGF	Involved in pancreatic beta cell development, vascularization, regeneration and differentiation. (Brissova et al., 2014)	VEGF signaling in pancreatic cancer can lead to malignant transformation of the pancreas when ligands bind with VEGFRs. Growth factors, genetic modifications, and hypoxia are some of the complicated processes that control the production of VEGF in tumor cells. (Nandy and Mukhopadhyay, 2011)	Sunitinib (Sutent): targets multiple receptor tyrosine kinases, including VEGFR, and has shown potential in reducing tumor growth and metastasis in pancreatic cancer (Doi et al., 2012)	combination of bevacizumab and gemcitabine (NCT00366457) and Axitinib (Inlyta) (NCT00219557) evaluated in treating pancreatic cancer (Waldner and Neurath, 2012; Duan et al., 2023; Srivani et al., 2018)
RAS-MAPK Pathway	Control cell cycle and differentiation. (Tidyman and Rauen, 2009). Insulin play a controlling function in the Ras-MAPK/ERK signaling pathway during INSR alternative splicing. (Malakar et al., 2016)	ERKs, JNKs, and p38MAPKs are the three separate effector classes that MAPKs belong to. JNKs are mainly involved in apoptosis and differentiation, p38MAPKs in stress responses, and ERKs in mitosis and proliferation (Furukawa, 2015)	CI-1040 and PD 0325901 showed promising effects in preclinical models, but clinical trials had mixed results. (Chappell et al., 2011; Adamopoulos et al., 2024; Li et al., 2022)	AB680 in Combination with AB122 Immunotherapy, Nab-Paclitaxel, and Gemcitabine (NCT04104672), Avutometinib (VS-6766) in combination with Gemcitabine and Nab-paclitaxel (NCT05669482) is in Phase I/II trial
PI3K-AKT-mTOR Pathway	AKT plays a role in cell survival and apoptosis by controlling the pro-survival and anti-apoptotic proteins Bcl-XL and NF-kB in both healthy and malignant cells. (Stanciu S et al., 2022)	RTKs activate PI3K signaling in pancreatic cancer, recruiting PI3Ks to phosphorylated tyrosine residues. PI3K binds via SH2 domain, activating its catalytic subunit allosterically. (Murthy et al., 2018)	LY294002 is a PI3K inhibitor inducing apoptosis *in vitro* and inhibiting tumor growth *in vivo*. Rapamycin inhibits mTOR kinase activity. (Chan et al., 2005	BA3011 (NCT03425279) is under trial, RADIANT-1 (NCT05669482) and Copanlisib (BAY 80–6946) (NCT02631590) drug being investigated for treating pancreatic cancer (Höpfner et al., 2008; Glaviano et al., 2023)
TGF- β	Diverse functions of TGF-β signaling are involved in the emergence, function, proliferation, death, and dedifferentiation of β cells. In both embryonic and mature β cells, TGF-β signaling usually inhibits the proliferation of β cells. (Wang et al., 2022)	TGF- β develops pancreatic cancer and treatment resistance through activating EMT. (Wang et al., 2017)	Targeting TGF- β signaling using small molecule inhibitors, monoclonal antibodies, or gene therapies are under investigation. (Liu et al., 2021)	Glipizide (NCT06168812) acts on hyperglycemia Galunisertib, Durvalumab (LY2157299) (NCT02734160) has shown promise in clinical trials. (Holmgaard et al., 2018; Kim et al., 2021)

## Conclusion

PC poses a significant difficulty because of its late-stage diagnosis and aggressive nature. Our complete investigation exposed the intricate interaction of oncogenes and risk factors, essential signaling networks in the development of the pancreas, and modulation in the same signaling pathway in PC. Our understanding of how transcription factors and signaling pathways are regulated in PC has grown by comprehending pancreas development. Despite having a low occurrence, genetic abnormalities and alterations in tumor suppressor genes significantly influence PC. Future research on precise mechanisms by which signaling pathways are activated and further exploration of the specific transcription factor expression patterns associated with PC could enable more personalized treatment strategies.

Different care strategies are used according to the stage and resectability of the cancer, with surgical procedures being explored in some situations. Despite difficulties, continuous research on signaling pathways and biomarkers can give optimism for improvements in early identification, more efficient therapies, and eventually better results for people with PC. Clinical trials, including combination therapies and signaling pathway inhibitors, hold promise for improving outcomes for PC.
